# Supplementary material for: Uncertainty-informed deep learning models enable high-confidence predictions for digital histopathology
Source: Nat Commun. 2022 Nov 2;13:6572. doi: 10.1038/s41467-022-34025-x (PMC9630455; doi:10.1038/s41467-022-34025-x)
Supplement: Supplementary file 1 — Supplemental Information [file 41467_2022_34025_MOESM1_ESM.pdf]

Supplementary Information for

Uncertainty-Informed Deep Learning Models Enable High-Confidence Predictions for Digital Histopathology

Contents

Supplementary Fig. 1

Supplementary Fig. 2

Supplementary Fig. 3

Supplementary Fig. 4

Supplementary Fig. 5

Supplementary Fig. 6

Supplementary Fig. 7

Supplementary Fig. 8

Supplementary Table 1

Supplementary Table 2

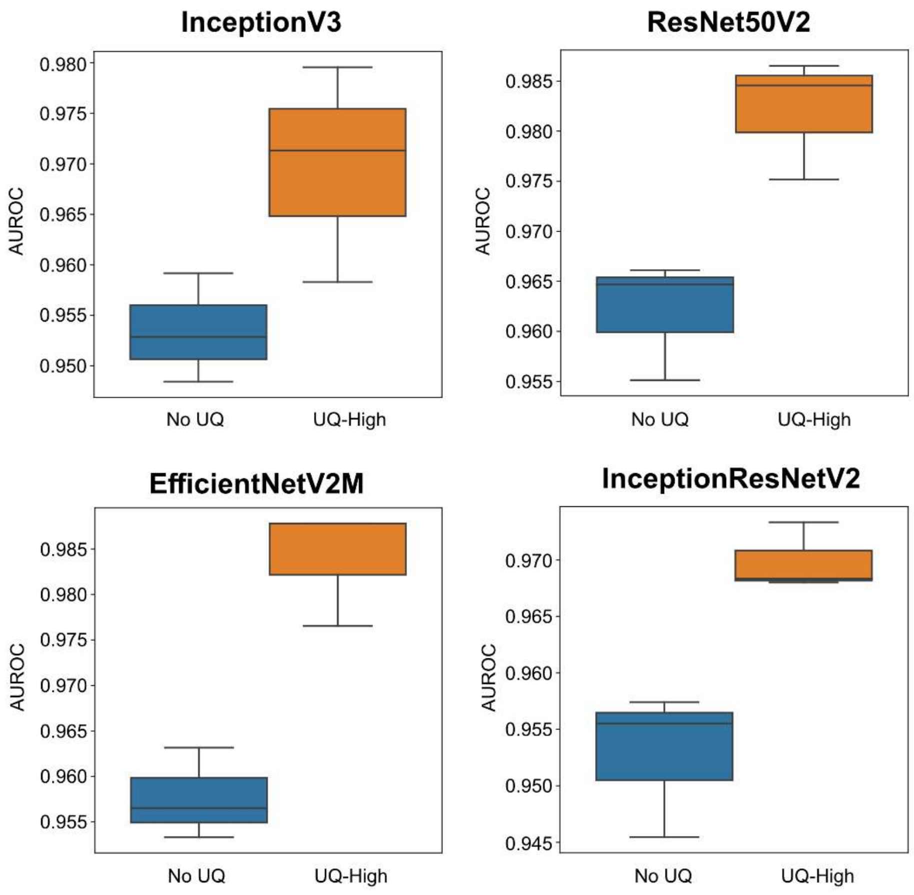

Supplementary Fig. 1. Models were trained in three-fold cross-validation at the maximum dataset size on TCGA data using four additional neural network architectures. The tested architectures include InceptionV3, ResNet50V2, EfficientNetV2M, and InceptionResNetV2, as implemented in Tensorflow/Keras. For each architecture, high-confidence predictions yield AUROCs higher than predictions from non-UQ models. Each boxplot summarizes AUROC from 6 trained models. For all boxplots, center line represents the median (50th percentile), lower and upper box bounds represent interquartile range (25th – 75th percentile), and minimum (lower whisker) and maximum (upper whisker) bounds extend to furthest datapoint up to 1.5 times the interquartile range, with outliers shown as diamonds.

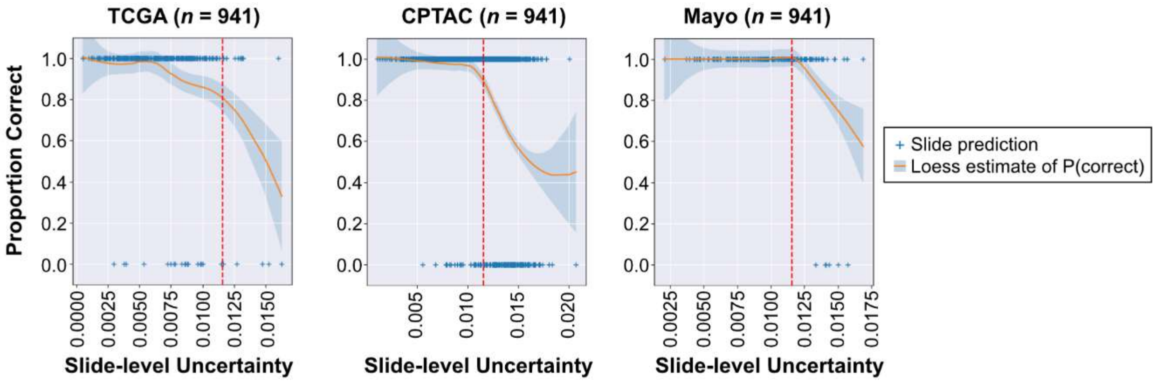

Supplementary Fig. 2. Association between slide-level uncertainty and misclassification. Slide-level uncertainty and classification accuracy was plotted for validation data from the TCGA cross-validation experiment at the maximum dataset size (left), and for results during evaluation of this model on the CPTAC and Mayo datasets (right). A Loess estimate of the probability of a correct diagnosis for each value of slide-level uncertainty is shown, with the shaded interval representing the 95% confidence interval obtained through bootstrapping. The red dotted line indicates the slide-level uncertainty threshold determined from nested cross-validation. In all cases, the probability of misclassification increases as uncertainty rises.

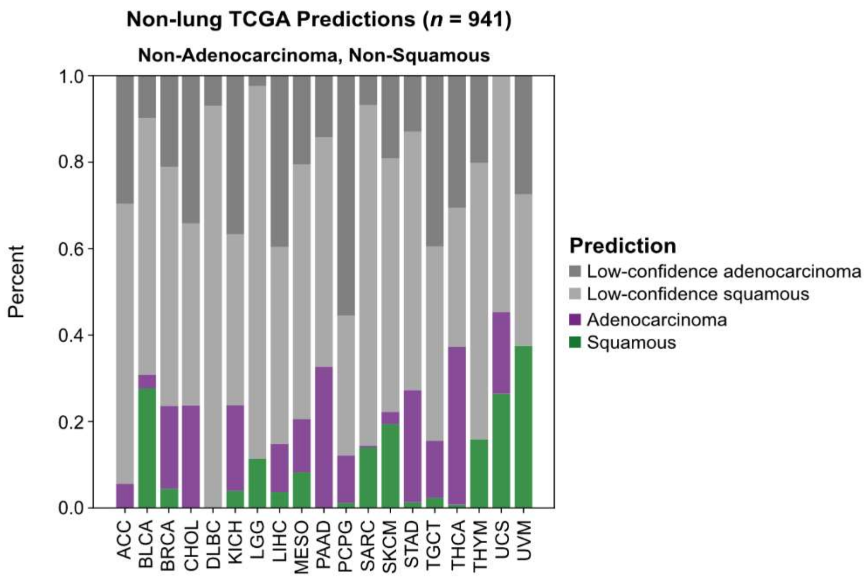

Supplementary Fig 3. Non-lung, out-of-distribution UQ predictions from a model trained on lung cancer. Predictions were generated for 4015 non-lung, non-adenocarcinoma, non-squamous cancers from TCGA. Cohorts with ≤5 total samples were removed from display. 3153 (78.5%) are reported as low confidence, with squamous cell predicted with high-confidence in 412 (10.3%) and adenocarcinoma predicted with high-confidence in 450 (11.2%).

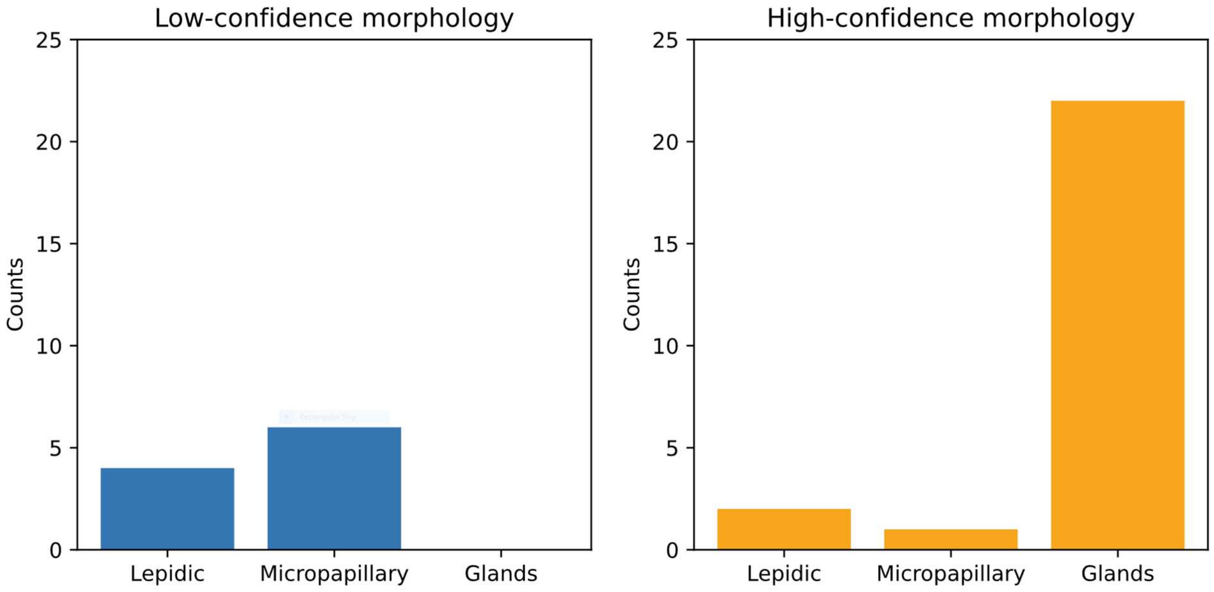

Supplementary Fig. 4. Quantitative assessment of pathologist-identified features among low- and high-confidence tiles from a whole-slide image. Two pathologists reviewed the 25 high-confidence and 25 low-confidence image tiles shown in **Fig 4**, with each tile classified as having lepidic morphology, micropapillary morphology, and/or clear glandular morphology. The number of low- and high-confidence image tiles showing each category of morphology is shown here.

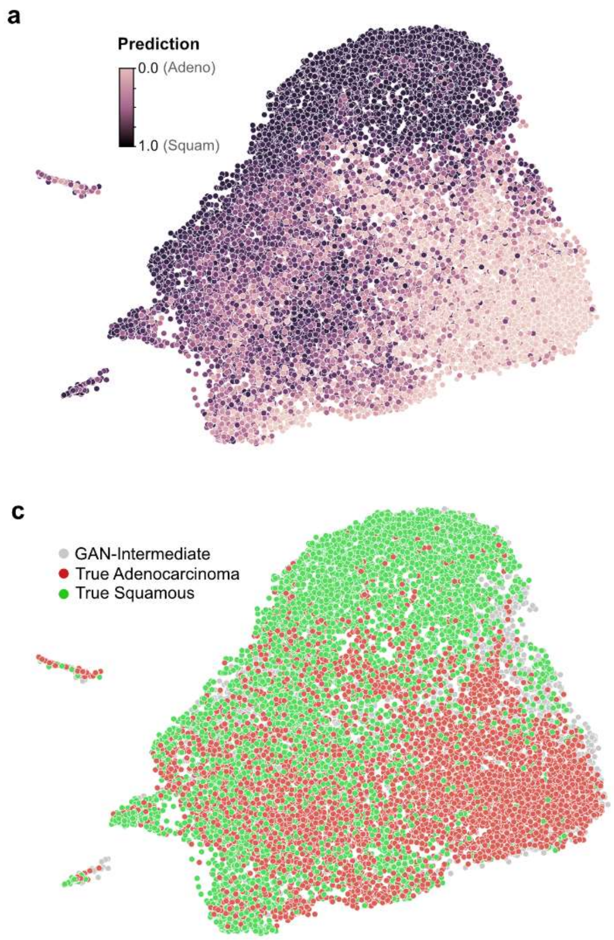

Supplementary Fig. 5. GAN-Intermediate slides approximate the LUAD-LUSC decision boundary. As with **Fig. 6**, penultimate layer activations were generated from a model trained on the full TCGA dataset for the CPTAC dataset and 1000 GAN-LUSC, GAN-LUAD, and GAN-Intermediate image tiles and plotted with UMAP. (**a**) Model predictions for each image tile are shown, scaled from 0 (adenocarcinoma) to 1 (squamous cell carcinoma). (**b**) Prediction uncertainty. (**c**) Images are labeled according to whether they are real slides from CPTAC or if they are one of the GAN-generated image cohorts. Here, it can be seen that the GAN-Intermediate image tiles are concentrated between GAN-LUSC and GAN-LUAD images and the adenocarcinoma/squamous cell prediction boundary.

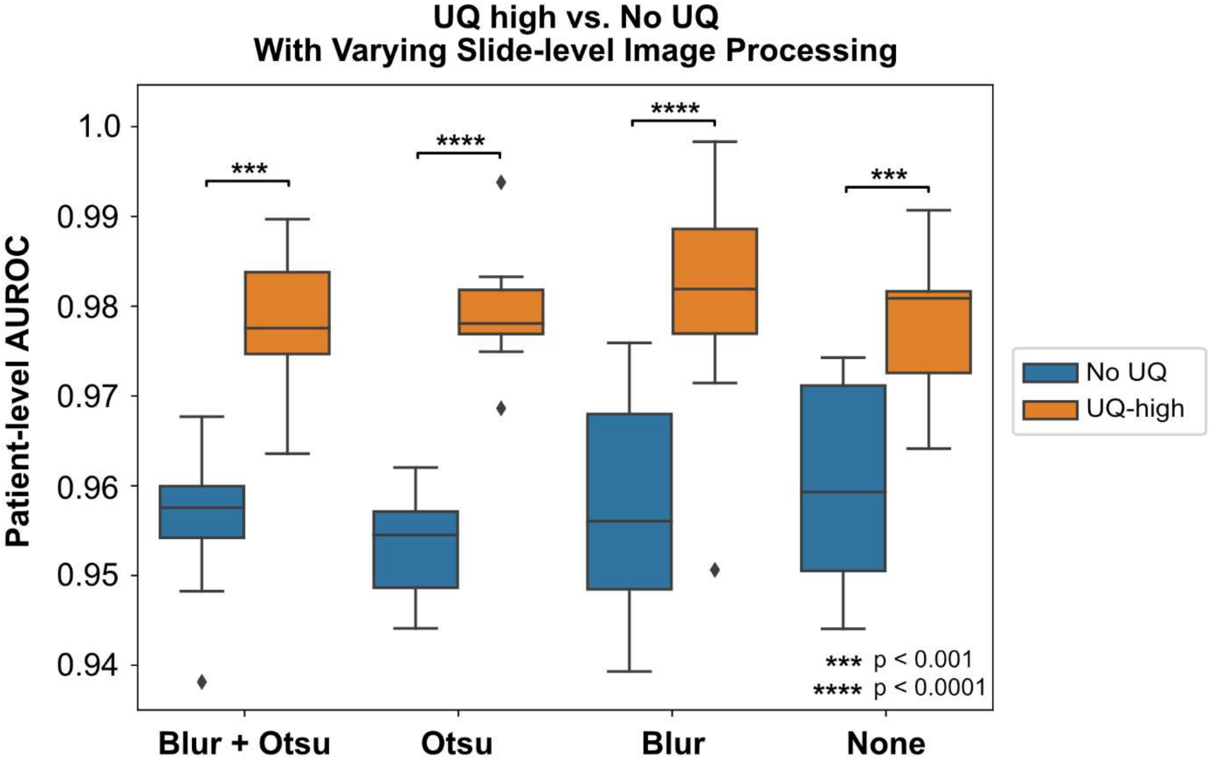

Supplementary Fig. 6. Uncertainty thresholding improves predictions regardless of slide-level background processing. Models were trained in three-fold cross-validation, bootstrapped three times, on the full TCGA training dataset with varying slide-level background and artifact filtering methods. We trained models either with Gaussian blur filtering and Otsu's thresholding, only Otsu's thresholding, only Gaussian blur filtering, or no background filtering method (only tile-level grayspace filtering). For each model, we trained models in five-fold nested cross-validation to determine uncertainty thresholds. In all cases, high-confidence UQ predictions as determined by thresholds from nested cross-validation outperformed predictions from models without UQ (Blur + Otsu: $p = 0.00018$, Otsu: $p < 0.0001$, Blur: $p < 0.0001$, None: $p = 0.00019$). Each boxplot summarizes AUROC from a total of 9 trained models. Statistical comparisons were performed using one-sided, paired *t*-tests without adjustment for multiple comparisons. For all boxplots, center line represents the median (50th percentile), lower and upper box bounds represent interquartile range (25th – 75th percentile), and minimum (lower whisker) and maximum (upper whisker) bounds extend to furthest datapoint up to 1.5 times the interquartile range, with outliers shown as diamonds.

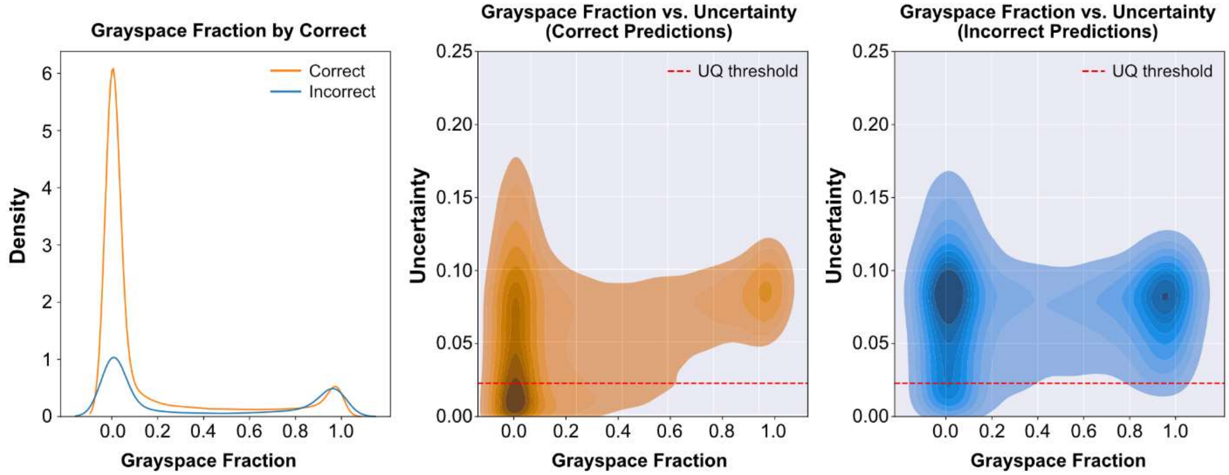

Supplementary Fig. 7. Assessment of the interaction between grayspace fraction and uncertainty. To investigate the potential impact of grayspace filtering on uncertainty quantification, we extracted all image tiles, without background filtering, from 50 lung adenocarcinomas and 50 lung squamous cell carcinomas in the CPTAC dataset. For each image tile, we calculated grayspace fraction, UQ-enabled model prediction, and estimated uncertainty. (**a**) Kernel density estimation for image tiles with varying grayspace fractions, separated by whether the prediction was correct or incorrect. There is a bimodal distribution of grayspace fraction in this dataset. Image tiles with low grayspace fraction (< 0.2) are more likely to be correctly predicted, and image tiles with high grayspace fraction (> 0.8) are just as likely to be correct as incorrect. (**b**) Two-dimensional kernel density estimation of grayspace fraction vs. uncertainty estimation for correctly predicted image tiles. When grayspace fraction is low, most correctly predicted image tiles fall below the uncertainty threshold and are thus classified as high-confidence. When grayspace fraction is high, most correct predictions fall above the uncertainty threshold and are thus filtered out as low-confidence. (**c**) Two-dimensional kernel density estimation of grayspace fraction vs. uncertainty estimation for incorrectly predicted image tiles. With high grayspace fraction, there is an increase in the number of incorrect predictions falling below the uncertainty threshold (erroneously classified as high-confidence) compared to correct predictions. These results support a grayspace fraction threshold of around 0.7 – 0.8 to maximize the utility of uncertainty estimation to enrich for correct predictions.

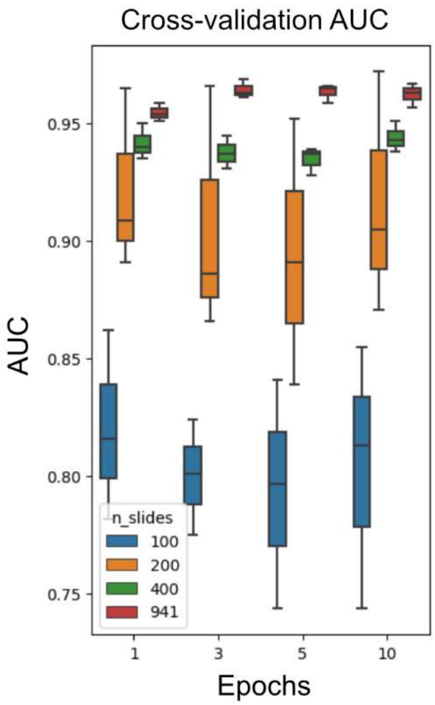

Supplementary Fig. 8. Epoch determination pilot experiment. Three-fold cross-validation was performed on the TCGA training dataset at a subsampled dataset size of 100, 200, 400, and 941 slides. Models were trained for 10 epochs, with performance recorded at epochs 1, 3, 5, and 10, in order to determine the optimal number of epochs for the rest of the experiment. As there was no significant improvement in cross-validated AUC beyond one epoch, all subsequent models were trained for one epoch. For all boxplots, center line represents the median (50th percentile), lower and upper box bounds represent interquartile range (25th – 75th percentile), and minimum (lower whisker) and maximum (upper whisker) bounds extend to furthest datapoint up to 1.5 times the interquartile range, with outliers shown as diamonds.

	TCGA		CPTAC		Mayo	
Characteristics	Adenocarcinoma	Squamous	Adenocarcinoma	Squamous	Adenocarcinoma	Squamous
Total patients	467	474	213	203	146	40
Males	215 (46.0%)	354 (74.7%)	132 (62.0%)	88 (43.3%)	62 (42.5%)	26 (65.0%)
Females	252 (54.0%)	120 (25.3%)	81 (38.0%)	22 (10.8%)	84 (57.5%)	14 (35.0%)
Not reported	0 (0%)	0 (0%)	0 (0%)	93 (45.8%)	0 (0%)	0 (0%)
Age (in years)						
Mean	65.2	67.3	62.5	65.8	69.5	67.7
Median	66	68	63	67	71	69
Range	33 – 88	39 – 90	25 – 81	40 – 88	49 – 91	30 – 92
Ancestry						
American Indian or Alaska Native	1 (0.2%)	0 (0%)	1 (0.5%)	0 (0%)	2 (1.4%)	0 (0%)
Asian	8 (1.7%)	9 (1.9%)	1 (0.5%)	0 (0%)	0 (0%)	0 (0%)
Black or African American	51 (10.9%)	29 (6.1%)	4 (1.9%)	1 (0.5%)	0 (0%)	0 (0%)
White	352 (75.4%)	326 (68.8%)	56 (26.3%)	32 (15.8%)	143 (97.9%)	40 (100%)
Not reported	55 (11.8%)	110 (23.2%)	151 (70.9%)	170 (83.7%)	1 (0.7%)	0 (0%)
Stage						
Stage I	254 (54.4%)	233 (49.2%)	107 (50.2%)	41 (20.2%)	104 (71.2%)	22 (55.0%)
Stage II	114 (24.4%)	153 (32.3%)	52 (24.4%)	44 (21.7%)	28 (19.2%)	11 (27.5%)
Stage III	66 (14.1%)	78 (16.5%)	48 (22.5%)	21 (10.3%)	14 (9.6%)	7 (17.5%)
Stage IV	25 (5.4%)	6 (1.3%)	3 (1.4%)	1 (0.5%)	0 (0%)	0 (0%)
Not reported	8 (1.7%)	4 (0.8%)	3 (1.4%)	96 (47.3%)	0 (0%)	0 (0%)
Slides per patient						
Mean	1	1	3.0	3.3	1.0	1
Median	1	1	3	3	1	1
Range	1	1	1 – 5	1 – 5	1 – 3	1
Total slides	467	474	644	662	150	40

Supplementary Table 1. Description of patient characteristics for the training and external validation datasets.

Hyperparameter / Model Parameter	Value
augment	xyrjb
batch_size	128
dropout	0.1
early_stop	TRUE
early_stop_method	accuracy
early_stop_patience	0
epochs	1
hidden_layer_width	1024
hidden_layers	2
include_top	FALSE
l1	0
l1_dense	0
l2	0
l2_dense	0
learning_rate	0.0001
learning_rate_decay	0.98
learning_rate_decay_steps	512
loss	sparse_categorical_crossentropy
model	xception
normalizer	reinhard_fast
optimizer	Adam
pooling	avg
tile_px	299
tile_um	302
toplayer_epochs	0
trainable_layers	0
training_balance	category
uq	TRUE
validation_balance	None

Supplementary Table 2. Deep learning model architecture and training hyperparameters.
