# Supplementary material for: Uncertainty-informed deep learning models enable high-confidence predictions for digital histopathology
Source: Nat Commun. 2022 Nov 2;13:6572. doi: 10.1038/s41467-022-34025-x (PMC9630455; doi:10.1038/s41467-022-34025-x)
Supplement: Supplementary file 3 — Description of additional Supplementary File [file 41467_2022_34025_MOESM3_ESM.pdf]

Descriptions of additional Supplementary Data files

- Supplementary Dataset 1: TCGA Diagnoses
- Supplementary Dataset 2: TCGA Slides
- Supplementary Dataset 3: CPTAC Diagnoses
- Supplementary Dataset 4: CPTAC Slides
- Supplementary Dataset 5: Mayo Diagnoses
- Supplementary Dataset 6: Mayo Slides
- Supplementary Dataset 7: Raw annotations for TCGA slides
- Supplementary Dataset 8: Raw annotations for CPTAC-LUAD slides
- Supplementary Dataset 9: Raw annotations for CPTAC-LSCC slides
- Supplementary Dataset 10: Raw annotations for Mayo slides
- Supplementary Dataset 11: Out of distribution diagnoses
