# Supplementary material for: Uncertainty-informed deep learning models enable high-confidence predictions for digital histopathology
Source: Nat Commun. 2022 Nov 2;13:6572. doi: 10.1038/s41467-022-34025-x (PMC9630455; doi:10.1038/s41467-022-34025-x)
Supplement: Supplementary file 15 — Reporting Summary [file 41467_2022_34025_MOESM15_ESM.pdf]

nature portfolio

Corresponding author(s): Alexander Pearson
Last updated by author(s): Sept 26, 2022

Reporting Summary

Nature Portfolio wishes to improve the reproducibility of the work that we publish. This form provides structure for consistency and transparency in reporting. For further information on Nature Portfolio policies, see our Editorial Policies and the Editorial Policy Checklist.

Statistics

For all statistical analyses, confirm that the following items are present in the figure legend, table legend, main text, or Methods section.

n/a	Confirmed	
☐	☒	The exact sample size (*n*) for each experimental group/condition, given as a discrete number and unit of measurement
☐	☒	A statement on whether measurements were taken from distinct samples or whether the same sample was measured repeatedly
☐	☒	The statistical test(s) used AND whether they are one- or two-sided<br>*Only common tests should be described solely by name; describe more complex techniques in the Methods section.*
☒	☐	A description of all covariates tested
☒	☐	A description of any assumptions or corrections, such as tests of normality and adjustment for multiple comparisons
☐	☒	A full description of the statistical parameters including central tendency (e.g. means) or other basic estimates (e.g. regression coefficient) AND variation (e.g. standard deviation) or associated estimates of uncertainty (e.g. confidence intervals)
☒	☐	For null hypothesis testing, the test statistic (e.g. *F*, *t*, *r*) with confidence intervals, effect sizes, degrees of freedom and *P* value noted<br>*Give P values as exact values whenever suitable.*
☒	☐	For Bayesian analysis, information on the choice of priors and Markov chain Monte Carlo settings
☒	☐	For hierarchical and complex designs, identification of the appropriate level for tests and full reporting of outcomes
☒	☐	Estimates of effect sizes (e.g. Cohen's *d*, Pearson's *r*), indicating how they were calculated

Our web collection on statistics for biologists contains articles on many of the points above.

Software and code

Policy information about availability of computer code

Data collection	No software was used for data collection.
Data analysis	All code necessary to reproduce the results of this manuscript, including the uncertainty thresholding algorithm, are provided at https://github.com/jamesdolezal/biscuit. Deep learning analyses were performed using Slideflow 1.1.0 (https://github.com/jamesdolezal/slideflow) with Tensorflow 2.7.1 as the primary deep learning package. This manuscript utilized QuPath 0.3.0 for region-of-interest pathologist annotations.

For manuscripts utilizing custom algorithms or software that are central to the research but not yet described in published literature, software must be made available to editors and reviewers. We strongly encourage code deposition in a community repository (e.g. GitHub). See the Nature Portfolio guidelines for submitting code & software for further information.

Data

Policy information about availability of data

All manuscripts must include a data availability statement. This statement should provide the following information, where applicable:

- Accession codes, unique identifiers, or web links for publicly available datasets
- A description of any restrictions on data availability
- For clinical datasets or third party data, please ensure that the statement adheres to our policy

Please email all requests for academic use of raw and processed data to the corresponding author. Whole-slide images from the The Cancer Genome Atlas (TCGA)

lung adenocarcinoma project are publicly available at https://portal.gdc.cancer.gov/projects/TCGA-LUSC, and images from the lung squamous cell project are available at https://portal.gdc.cancer.gov/projects/TCGA-LUAD. Whole-slide images from the Clinical Proteomic Tumor Analysis Consortium (CPTAC) lung adenocarcinoma and lung squamous cell carcinoma collections are available at https://www.cancerimagingarchive.net/collections/, under the collection names "CPTAC-LUAD" and "CPTAC-LSCC", respectively. TCGA and CPTAC slide identifiers for images used in this study are included in the supplementary information. Restrictions apply to the availability of the internal Mayo Clinic dataset, which was used with institutional permission for the current study and is thus not publicly available due to data privacy laws. All requests will be promptly evaluated based on institutional and departmental policies to determine whether the data requested are subject to intellectual property or patient privacy obligations. Data can only be shared for non-commercial academic purposes and will require a data user agreement. Source data are provided with this paper.

Human research participants

Policy information about studies involving human research participants and Sex and Gender in Research.

Reporting on sex and gender	N/A
Population characteristics	N/A
Recruitment	N/A
Ethics oversight	N/A

Note that full information on the approval of the study protocol must also be provided in the manuscript.

Field-specific reporting

Please select the one below that is the best fit for your research. If you are not sure, read the appropriate sections before making your selection.

☒ Life sciences ☐ Behavioural & social sciences ☐ Ecological, evolutionary & environmental sciences

For a reference copy of the document with all sections, see nature.com/documents/nr-reporting-summary-flat.pdf

Life sciences study design

All studies must disclose on these points even when the disclosure is negative.

Sample size	Sample sizes were chosen based on the maximum available dataset size for public repositories (TCGA and CPTAC) and the maximum obtainable sample size for the institutional dataset (Mayo Clinic).
Data exclusions	Data exclusions are detailed in the Methods section of the main manuscript. For the primary deep learning models, any sample without a diagnosis of lung adenocarcinoma or squamous cell carcinoma was excluded
Replication	The primary code repository (https://www.github.com/jamesdolezal/biscuit) contains all code necessary for replication of all experimental results.
Randomization	Randomization was not applicable to this study, as this study did not involve a clinical intervention.
Blinding	Blinding was not applicable to this study, as this study did not involve a clinical intervention.

Reporting for specific materials, systems and methods

We require information from authors about some types of materials, experimental systems and methods used in many studies. Here, indicate whether each material, system or method listed is relevant to your study. If you are not sure if a list item applies to your research, read the appropriate section before selecting a response.

Materials & experimental systems

n/a	Involved in the study
☒	☐ Antibodies
☒	☐ Eukaryotic cell lines
☒	☐ Palaeontology and archaeology
☒	☐ Animals and other organisms
☒	☐ Clinical data
☒	☐ Dual use research of concern

Methods

n/a	Involved in the study
☒	☐ ChIP-seq
☒	☐ Flow cytometry
☒	☐ MRI-based neuroimaging
